# Telesimulation Training for Endoscopic Mitral Valve Surgery: An Air-Pilot Training Concept for Distance Training

**DOI:** 10.1177/15569845241237778

**Published:** 2024-04-04

**Authors:** Shokoufeh Cheheili Sobbi, Umar Imran Hamid, Arian Arjomandi Rad, Marvin Fillet, Jos Maesen, Peyman Sardari Nia

**Affiliations:** 1 Department of Cardiothoracic Surgery, Heart and Vascular Centre Maastricht University Medical Centre, The Netherlands; 2 Maastricht University, The Netherlands

**Keywords:** simulation, endoscopic mitral valve surgery, mitral valve repair, mitral valve surgery, telesimulation

## Abstract

**Objective:**

The aim of this study was to validate and assess the feasibility and impact of telesimulation training on surgical skills using a portable mitral valve telesimulator.

**Methods:**

A telesimulation course composed of 3 online modules was designed based on backwards chaining, preassessment and postassessment, performance feedback, hands-on training on a telesimulator, and the theoretical content. A fully 3-dimensional–printed and transportable telesimulator was developed and sent out to the participants with instruments that were needed. Feedback about the platform was obtained from participants to validate its value as a training tool. Theoretical and technical assessments were carried out before and after the course. Technical assessments were based on the accuracy and time taken to place sutures at the anterior and posterior mitral annulus.

**Results:**

In total, 11 practicing cardiac surgeons from Oceania, Asia, Europe, and North America completed the course. Theoretical preassessment and postassessment showed that participants scored significantly higher on postassessment (mean 87.5% vs 68.1%, *P* < 0.004). The participant evaluation scores of the simulator as a tool for endoscopic mitral valve surgery was 4 to 5 out of 5. There was a significant improvement in the speed (median 14.5 vs 39.5 s, *P* < 0.005) and the accuracy to place sutures in the mitral valve annulus following course completion (*P* < 0.001).

**Conclusions:**

Here we validated the educational value of a novel telesimulation platform and validated the feasibility to teach participants at a distance the knowledge and skills for endoscopic mitral valve surgery. Future studies will be required to validate the improvement in skills during surgery.

## Graphical abstract

**Visual abstract:**
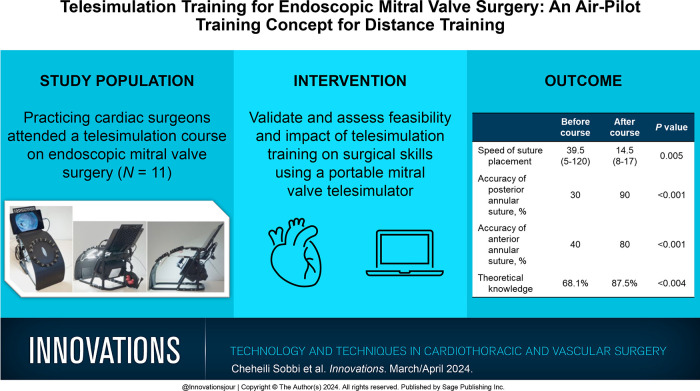

**Central Message**

We have validated the educational value of a telesimulation platform and the feasibility of teaching participants at a distance the knowledge and skills for endoscopic mitral valve surgery. The telesimulation training course was completed by 11 practicing cardiac surgeons from around the world.

## Introduction

The change in work patterns and patient safety and the increase in the complexity of cardiac surgical procedures have forced the development of models that have moved training from the operating room to a simulation platform. Earlier research has highlighted the importance of simulation training and showed that a short period of focused training could expedite the acquisition of technical skills.^1–6^ A reduction in time to achieve proficiency has been shown when simulation training is incorporated.^1^ The importance of simulation training on surgical skills for minimally invasive surgery has also been shown before.^7^

We have developed a high-fidelity minimally invasive mitral simulator and validated the platform previously.^8^ Furthermore, we have validated the educational value of our simulator in the 2-day in-person simulation training course and showed that it significantly improves endoscopic skills.^9^ The COVID-19 pandemic has resulted in a shift from traditional to online teaching.^10^ The effectiveness of distance learning on surgical training has been evaluated in past studies, and the lack of hands-on training seemed to be a point of concern.^10^ The aim of the study was to validate and assess the feasibility and impact of telesimulation training on surgical skills using a portable mitral valve telesimulator.

## Methods

### Telesimulation Platform

**Fig. 1.**
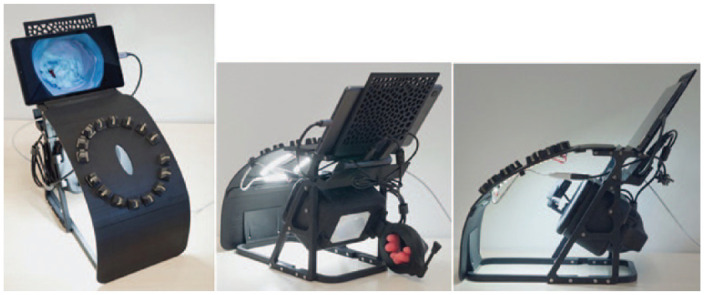
Different views of the telesimulation platform.

**Fig. 2.**
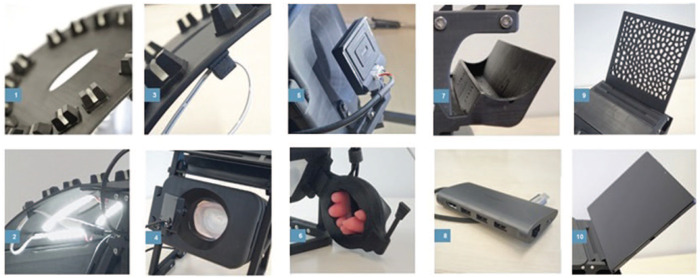
Components of the telesimulation platform. (1) Chest, (2) LED array, (3) LED array cable holder, (4) central box, (5) USB camera, (6) papillary muscle holder, (7) USB-C hub holder, (8) USB-C multiport hub, (9) mobile device holder, (10) mobile device (tablet). LED, light-emitting diode; USB, universal serial bus.

A fully 3-dimensional (3D)–printed and transportable telesimulator was developed (Fig. 1, Fig. 2). The model was created with computer-aided board design software and printed with a digital light processing 3D printer and a postcuring machine. The platform has a central chest plate with surrounding guides for suture placement. The center of the chest plate has an opening to simulate a utility port. Furthermore, the platform has an inner module composed of a 3D-printed left ventricle and atrium with fittings for disposable mitral and papillary muscles. The mitral valve and papillary muscles are disposable, providing the opportunity to be trained in repairing different mitral valve pathologies. The manufacturing of the silicone valve replica is described elsewhere.^11^ The device is provided with a universal serial bus (USB)–C hub connected to a USB charger, to power a smart device, light-emitting diode array, and USB camera. The telesimulator could be connected to any smart device and provide live connectivity. The hub provides the image stream to the tablet on which the valve footage is displayed for simulation and for recording.

### Telesimulation Course

A telesimulation course composed of 3 online modules was designed based on backwards chaining, preassessment and postassessment, performance feedback, hands-on training on telesimulator, and the theoretical content. In our course, we employed the backward chaining learning strategy, which is proven to be more efficient in education and training programs. Backward chaining is a strategy that involves starting from a desired learning objective and working backward to determine the sequence of steps and actions needed to achieve these goals. First, the learning objectives are defined and pretraining assessment is developed to determine whether the objectives are met. This strategy specially programs a high success-to-failure ratio and has shown to be effective in surgical simulation trainings.^12,13^ Then, one determines how to execute the learning and then creates the course content. The basic learning objectives for our course were as follows:

- Making the participants aware of necessary skills, knowledge, organization, and teamwork for starting the endoscopic mitral valve program and evaluate these through preassessment and postassessment.
- Acquiring theoretical knowledge focused on starting an endoscopic mitral program to evaluate these through preassessment and postassessment.
- Acquiring endoscopic skills with long-shafted instruments for mitral valve repair, namely, annuloplasty suturing.

**Fig. 3.**
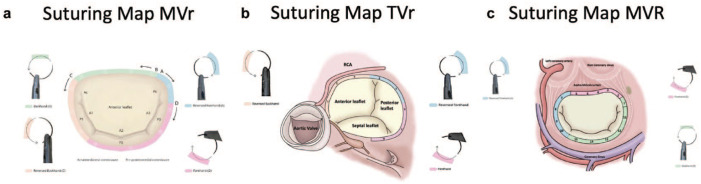
Suturing maps for endoscopic MV and TV surgery.^15–17^ (a) Endoscopic MVr, (b) TVr, and (c) MVR. MVr, mitral valve repair; MVR, mitral valve replacement; TVr, tricuspid valve repair.

Two telesimulation courses were designed and organized in March and December of 2022. The platform was provided to 11 practicing cardiac surgeons from Oceania, Asia, Europe, and North America. Participants registered for the open training via Heart Team Academy and were selected randomly.^14^ None of the participants had any conflict of interest with the inventor. All course materials including the telesimulator and long-shafted instruments were sent to the trainees by mail, and they were returned after the training. The course was designed following the philosophy of air-pilot training, in which participants initially acquire knowledge and skills through rigorous theoretical and practical learning sessions, along with assessments, before gaining real-time experience. Each course was composed of 3 online modules, each lasting 2.5 h. Each module was composed of a theoretical part covering various aspects of starting an endoscopic mitral valve surgery program and a hands-on part on the telesimulator. In addition, an online resource in the form of suturing map was also provided to the participants so that they could become familiar with needle angles for suturing in different areas of the mitral valve annulus (Fig. 3).^15^

### Telesimulation Studio

**Fig. 4.**
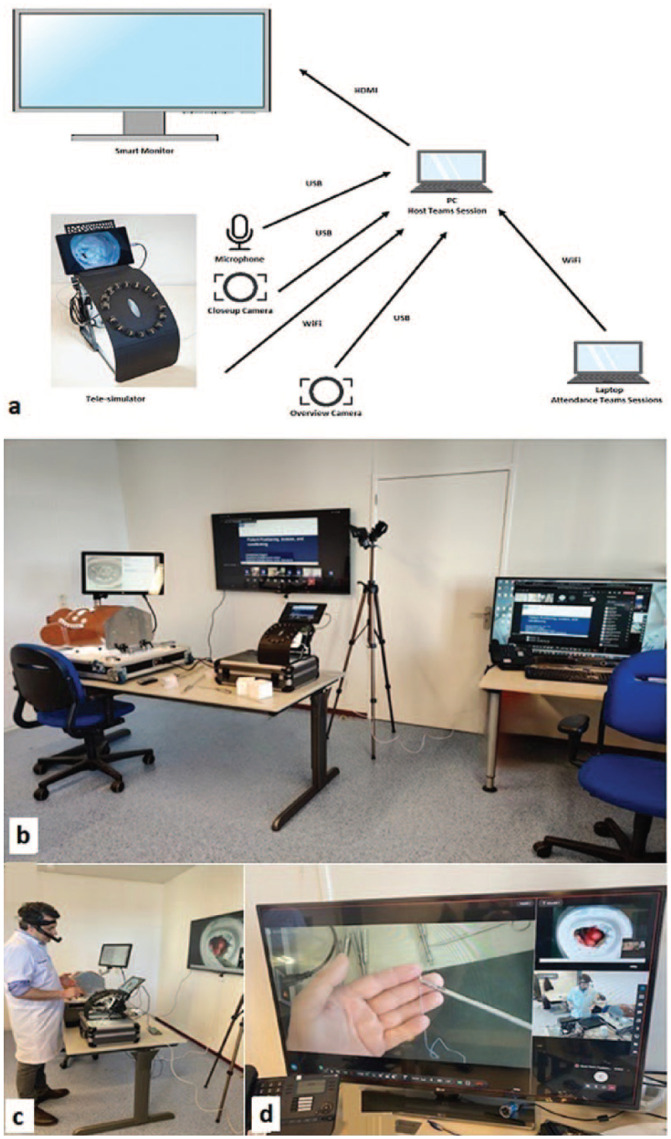
Telesimulation infrastructure. (a) Schematic view and (b) (c) (d) pictures of the telesimulation studio. HDMI, high-definition multimedia interface; PC, personal computer; USB, universal serial bus.

A telesimulation studio was designed to facilitate live streaming and on-distance guiding of the participants (Fig. 4). The infrastructure of the studio was composed of the following:

- 1 personal computer (PC) of host for team session
- 1 laptop for PowerPoint presentations for attendees of team sessions
- 1 telesimulator with live connectivity via WiFi to the PC
- 1 camera that was connected via USB to the PC, which provided closeup images of the surgical instruments, particularly the needle angle in the needle holder
- 1 camera that was connected to the PC for the overall view
- 1 microphone that was connected via USB to the PC
- 1 central smart monitor

The streams of all connected equipment were merged or isolated by software according to the desired image. This new stream was then cast via Microsoft Teams to the candidates.

### Validation of Telesimulation Platform

A questionnaire-based assessment of the platform on the 1- to 5-point Likert scale was completed by participants to assess the level of agreement with the 6 statements (Table 1).^15^

**Table 1.** Simulator Feedback.

Statement	Response^a^
The mitral valve in the simulator looked realistic.	4.5 (4–5)
The mitral valve in the simulator felt realistic.	4 ( 4–5)
The relation of the mitral valve to the thorax was realistic.	5 (4–5)
Performing sutures on the valve felt realistic.	4 (4–5)
The simulator is a good method for training minimally invasive mitral valve repair.	5
I am more aware of the skills needed to perform minimally invasive mitral valve repair.	5

Data are reported as median (range).

a Response values: 1 = *strongly disagree*, 2 = *disagree*, 3 = *neither disagree nor agree*, 4 = *agree*, and 5 = *strongly agree*.

### Validation of Educational Value of Telesimulation

A technical and theoretical (fundemtal knowlegde related to endoscopic mitral valve surgery) assessment was carried out before and after the course. The technical assessments were based on the accuracy of suture placement, which required the needle to enter and exit the mitral valve annulus using endoscopic instruments. Suture accuracy was determined by scoring the entrance and exit in the mitral valve annulus. If both the entrance and the exit of the suture were correctly placed, then the suture was scored as accurate. The time taken to place a suture at the anterior and posterior annulus of the silicone mitral valve was also recorded. The technical preassessment was performed without prior practice on the simulator. The technical postassessment was performed in the last module of each course. All technical assessments were recorded and retrospectively scored by 2 independent and anonymized observers. The assessments were based on a continuous observation of the videos.

### Ethical Approval

Approval was obtained from the medical ethics committee, and all course participants consented prospectively to participate in the study.

### Statistical Analysis

The data were analyzed using GraphPad Prism software version 5 (GraphPad Software, Boston, MA, USA). Paired measurements were subject to the Wilcoxon signed-rank test to assess potential differences between measurements. Fisher’s exact test was used to assess the contingency tables. Variables were presented as frequencies and percentages, mean ± standard deviation, or median with the 25th and 75th percentiles in the presence of skewness. A *P* value <0.05 was considered statistically significant.

## Results

### Validation of the Platform

All participants completed all 3 modules within the course. The participant evaluation scores were 4 to 5 out of 5 (Table 1). The participants agreed that the simulator looked realistic and the silicone mitral valve and suturing experience felt realistic (score 4 to 5 out of 5). The participants also agreed that the platform is an excellent tool for minimally invasive mitral valve surgery (score 5 out of 5).

### Validation of Educational Value of Telesimulation

**Fig. 5.**
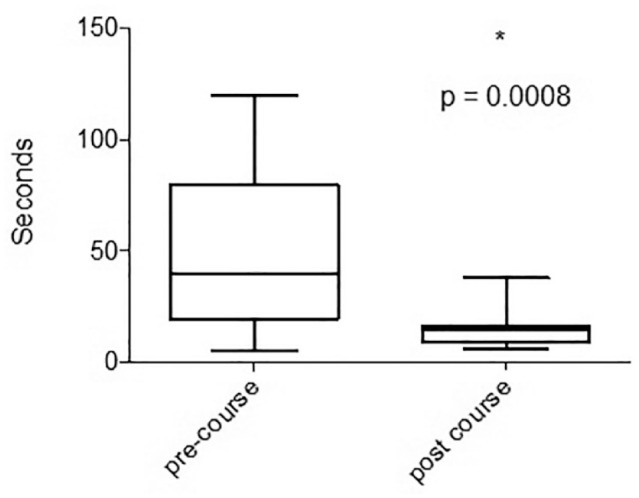
The time taken for suture placement before and after course completion. Box and whisker plots demonstrate reduction in the time taken for suture placement.

Theoretical preassessment and postassessment showed that participants scored significantly higher on the postassessment (mean 87.5% vs 68.1%, *P* < 0.004). There was a significant improvement in the accuracy of annular suture placement (anterior, 30% vs 90%; posterior, 40% vs 80%) following completion of the course (*P* < 0.001). Similarly, there was also a significant reduction in the time taken to place sutures (39.5 vs 14.5 s) at the anterior and posterior mitral valve annulus, before and after course completion (*P* < 0.001; Table 2, Fig. 5).

**Table 2.** Technical Course Assessment.

Technical course assessment	Before course	After course	*P* value
Time taken for annular suture placement (s)	39.5 (5–120)	14.5 (8–17)	0.005^a^
Accuracy posterior annular suture	30	90	<0.001^b^
Accuracy anterior annular suture	40	80	<0.001^b^

Data are reported as median (range) or %.

a Wilcoxon signed-rank test

b Fisher’s exact test

## Discussion

The importance of simulation-based training for learning new surgical skills has been established in our previous research.^9,11^ Surgical training involves both theoretical and practical components; however, there is a lack of validation and standardization of simulation training within the surgical curriculum. Although simulation training is still relatively new, there is a substantial amount of evidence demonstrating its impact on the acquisition of surgical skills.^1–6^

Various different types of simulation training models are currently in use, including task deconstruction models, human cadaver models, 3D-printed models, and virtual reality simulation, each with its own strengths and weaknesses.^11,18^ Similarly, we have put forward pivotal points for an ideal simulator, which should be a reusable, portable, and realistic suturing experience; provide haptic feedback; and incorporate multiple surgical approaches and user interfaces.^8,11^ A minimally invasive mitral valve surgery simulator has proven to improve endoscopic surgical skills for mitral valve repair after a 2-day course.^9^

Our current telesimulation platform, as described in this study, is based on the key elements stated above and can easily be transported to various locations for training. The main advantages of telesimulation training are that the training would be accessible to more surgeons, omitting the need for distance travel and reducing the subsequent costs. Additional to this, the participants would have the simulator device during a period for continuous practice outside the course.

The ability to record the simulation training exercises allows for objective assessments on suture placement, needle angles, movements, and so forth by different assessors or a possible motion-detection program that could enhance our knowledge of surgical education.

Our telesimulation provides the possibility to be trained in repairing different mitral valve pathologies by simulation of various mitral valve repair techniques such as resection, placement of neochords, and annuloplasty ring implantation.

The current study has several limitations. Our study focuses on annular suture placements. However, this is only 1 part of the mitral valve repair process. This study did not analyze other steps involved in endoscopic mitral valve repair. In addition, we do not know if this telesimulation training has any impact on surgical skills during real endoscopic mitral valve repair. Future research is recommended to assess the impact of training with telesimulation on surgical skills during actual surgeries in the operating theater.

## Conclusions

We have validated the educational value of a novel telesimulation platform and the feasibility of teaching participants at a distance the knowledge and skills for endoscopic mitral valve surgery. Further studies will be required to objectively verify the effect of training on this platform and the impact on the learning curve for endoscopic mitral valve surgery. Then, this telesimulation training could be offered worldwide in the future.
